# The Antiviral Effects of the Symbiont Bacteria *Wolbachia* in Insects

**DOI:** 10.3389/fimmu.2020.626329

**Published:** 2021-01-29

**Authors:** André C. Pimentel, Cássia S. Cesar, Marcos Martins, Rodrigo Cogni

**Affiliations:** Department of Ecology, University of São Paulo, São Paulo, Brazil

**Keywords:** antiviral, *Wolbachia*, insects, arboviruses, evolution, wild populations, review, endosymbiont

## Abstract

*Wolbachia* is a maternally transmitted bacterium that lives inside arthropod cells. Historically, it was viewed primarily as a parasite that manipulates host reproduction, but more recently it was discovered that *Wolbachia* can also protect *Drosophila* species against infection by RNA viruses. Combined with *Wolbachia’*s ability to invade insect populations due to reproductive manipulations, this provides a way to modify mosquito populations to prevent them transmitting viruses like dengue. In this review, we discuss the main advances in the field since *Wolbachia’s* antiviral effect was discovered 12 years ago, identifying current research gaps and potential future developments. We discuss that the antiviral effect works against a broad range of RNA viruses and depends on the *Wolbachia* lineage. We describe what is known about the mechanisms behind viral protection, and that recent studies suggest two possible mechanisms: activation of host immunity or competition with virus for cellular resources. We also discuss how association with *Wolbachia* may influence the evolution of virus defense on the insect host genome. Finally, we investigate whether the antiviral effect occurs in wild insect populations and its ecological relevance as a major antiviral component in insects.

## Introduction

*Wolbachia pipientis* is a maternally transmitted alphaproteobacterium that lives obligatorily within the cytoplasm of arthropod cells (1). Until recently it was viewed primarily as a parasite that manipulates host reproduction, most commonly by inducing cytoplasmic incompatibility (2, 3). Cytoplasmic incompatibility allows *Wolbachia* to invade insect populations by causing embryonic mortality when uninfected females mate with infected males, thus conferring a selective advantage to infected females (4, 5). In 2008, two studies discovered that *Wolbachia* can protect *Drosophila melanogaster* against RNA viruses (6, 7). Subsequently, it was discovered that *Wolbachia* can block dengue virus replication in mosquitoes (8, 9). These findings provided a new way in which *Wolbachia* can be used to control human arboviruses, since previous attempts relied on using cytoplasmic incompatibility as a transgene driver, or reduction of mosquito longevity by a virulent *Wolbachia* strain. *Wolbachia* lineages from different insects that were transferred to the mosquito *Aedes aegypti* can limit the replication of arboviruses such as Dengue virus (DENV), Chikungunya virus (CHIKV), Yellow Fever virus (YFV), Zika virus (ZIKV) and West Nile virus (WNV) (9–12). *Wolbachia* can spread quickly through mosquito populations by cytoplasmic incompatibility (13–15), and large field trials have been successful in reducing dengue prevalence in human populations (16, 17).

In this Mini Review, we discuss the main advances in the field since the *Wolbachia* antiviral effect was discovered 12 years ago, current research gaps, and potential future developments. First, we address the generality of the antiviral effect and how it depends on *Wolbachia* lineage and on virus identity. Second, we discuss the possible mechanisms of antiviral protection. Third, we discuss how association with *Wolbachia* may influence the evolution of virus defense on the insect host genome. Finally, we discuss the virus blocking ecological relevance by addressing if it occurs in wild insect populations.

## Generality: Different Viruses and Different *Wolbachia* Lineages

After the first studies showing that *Wolbachia* protects flies and mosquitoes against RNA viruses (6–8) and its potential to control insect-born human diseases (8–10, 14), there was a great interest in the area. Many studies conducted on mosquitoes tested for their vector competence and revealed that *Wolbachia* reduces infection and, in some cases, the dissemination and transmission of diseases such as dengue, chikungunya, yellow fever, zika, and West Nile fever (**Table 1**). In flies, *Wolbachia* protects mostly against Flock House virus (FHV), and *Drosophila* C virus (DCV). However, DCV is not commonly found in wild *Drosophila* populations (41) and there is limited information on protection against viruses that are common in nature, such as Nora (6) and Kallithea virus (36) (**Table 1**). Although many studies report *Wolbachia* protection against different viruses, there are a few cases in which *Wolbachia* provides no protection or even increases the host susceptibility to viral infection (**Table 1**). Furthermore, only three studies investigated *Wolbachia* protection against DNA viruses (6, 36, 40) and none found evidence of protection (**Table 1**). Therefore, *Wolbachia* protection in insects is a general phenomenon only against RNA viruses.

**Table 1:** *Wolbachia* antiviral effect on insects.

*Wolbachia* effect	*Wolbachia* strain	Natural host species	Tested host species	Tested virus	Study
Protection	*w*AlbB	*Aedes albopictus*	*Aedes aegypti, Aedes polynesiensis*	DENV, SFV, ZIKV	Bian et al., 2010^b^ (8), Bian et al., 2013^b^ (18), Ant et al., 2018^b^ (19), Joubert et al., 2016^b^ (20)
	*w*AlbB + *w*Mel	*Aedes albopictus + Drosophila melanogaster*	*Aedes aegypti*	DENV	Joubert et al., 2016^b^ (20)
	*w*AlbA + *w*AlbB	*Aedes albopictus*	*Aedes albopictus*	DENV	Mousson et al., 2012^b,e^ (21)
	*wC. quinquefasciatus*	*Culex quiquenfasciatus*	*Culex quiquenfasciatus*	WNV	Glaser & Meola, 2010^b^ (12)
	*w*Ana	*Drosophila ananassae*	*Drosophila simulans*	DCV, FHV	Martinez et al., 2014^a,b^ (22), Martinez et al., 2017^a^ (23)
	*w*Ara	*Drosophila arawakana*	*Drosophila simulans*	DCV, FHV	Martinez et al., 2014^a,b^ (22)
	*w*Au	*Drosophila simulans*	*Aedes aegypti, Drosophila simulans*	DENV, ZIKV, SFV, DCV, FHV	Ant et al., 2018^b^ (19), Martinez et al., 2014^a,b^ (22), Martinez et al., 2017^a,b^ (23), Osborne et al., 2009^a,b^ (24)
	*w*Ha	*Drosophila simulans*	*Drosophila simulans*	DCV, FHV	Martinez et al., 2017^a^ (23), Osborne et al., 2009^a^ (24)
	*w*Mel	*Drosophila melanogaster*	*Aedes aegypti, Aedes albopictus, Drosophila simulans, Drosophila melanogaster*	CHIKV, DCV, DENV, FHV, Flavivirus OTU2, ZIKV, SFV, WNV	Amuzu et al., 2018^b^ (25), Ant et al., 2018^b^ (19), Blagrove et al., 2012^b^ (26), Martinez et al., 2014^a,b^ (22), Fraser et al., 2017^b^ (27), Hussain et al., 2013^b,f^ (28), Joubert et al., 2016^b^ (20), Martinez et al., 2017^a,b^ (23), Osborne et al., 2009^a,b^ (24), Van den Hurk et al., 2012^b,c,e,g^ (10), Walker et al., 2011^b,c^ (14), Ye et al., 2016^b,c,e^ (29), Rancés et al., 2012^b^ (30)
	*w*MelCs	*Drosophila melanogaster*	*Aedes aegypti, Drosophila simulans, Drosophila melanogaster*	CHIKV, CrPV, DCV, DENV, FHV, WNV	Martinez et al., 2014^a,b^ (22), Hedges et al., 2008^a^ (7), Fraser et al., 2017^b^ (27), Hussain et al., 2013^b^ (28), Martinez et al., 2017^a,b^ (23), Glaser & Meola, 2010^b^ (12)
	*w*MelPop	*Drosophila melanogaster*	*Aedes aegypti, Drosophila melanogaster*	CHIKV, DCV, DENV, FHV, Nora virus, YFV	Hedges et al., 2008^a^ (7), Joubert et al., 2016^b^ (20), Martinez et al., 2017^a,b^ (23), Teixeira et al., 2008^a,b^ (6), Van den Hurk et al., 2012^b,c,e,h,i^ (10), Walker et al., 2011^b,c^ (14), Moreira et al., 2009^b,c^ (9)
	*w*Stv	*Drosophila sturtevanti*	*Drosophila simulans*	DCV	Martinez et al., 2014^a,b^ (22)
	*w*Tei	*Drosophila teissieri*	*Drosophila simulans, Drosophila teissieri*	DCV, FHV	Martinez et al., 2014^a,b^ (22), Martinez et al., 2017^a^ (23)
	*w*Tro	*Drosophila tropicalis*	*Drosophila simulans, Drosophila tropicalis*	DCV, FHV	Martinez et al., 2014^a^ (22), Martinez et al., 2017^a,b^ (23)
	*w*Ma	*Drosophila simulans*	*Drosophila simulans*	FHV	Martinez et al., 2014^a^ (22), Martinez et al., 2017^a^ (23)
	*w*Ri	*Drosophila simulans*	*Aedes aegypti, Drosophila simulans*	DCV, DENV, FHV	Fraser et al., 2017^b^ (27), Martinez et al., 2017^a^ (23), Osborne et al., 2009^a^ (24)
	*w*Pro	*Drosophila prosaltans*	*Drosophila prosaltans, Drosophila simulans*	FHV	Martinez et al., 2017^a^ (23)
	*w*Yak	*Drosophila yakuba*	*Drosophila simulans*	FHV	Martinez et al., 2014^b^ (22)
	*w*Inn	*Drosophila innubila*	*Drosophila innubila*	FHV	Unckless and Jaenike et al., 2012^a^ (31)
	*w*Suz	*Drosophila suzukii*	*Drosophila suzukii*	DCV, FHV	Cattel et al., 2016^a,b,d^ (32)
	*w*Stri	*Laodelphax striatellus*	*Nilaparvata lugens*	RRSV	Gong et al., 2020^b^ (33)
No protection	*w*Pip	*Culex pipiens*	*Culex pipiens*	CpVD	Altinli et al., 2019^b^ (34)
	*w*Noto	*Aedes notoscriptus*	*Aedes notoscriptus*	DENV	Skelton et al., 2016^b,c^ (35)
	*w*Mel	*Drosophila melanogaster*	*Aedes aegypti, Drosophila melanogaster, Drosophila simulans*	CHIKV, DENV, Flavivirus OTU1, Flavivirus OTU3, Flavivirus OTU16, Flavivirus OTU25, Flavivirus OTU20, Flavivirus OTU21, FHV, ZIKV, WNV, YFV	Amuzu et al., 2018^b^ (25), Ant et al., 2018^b^ (19), Martinez et al., 2014^b^ (22), Martinez et al., 2017^b^ (23), Hussain et al., 2013^b,f^ (28), Van den Hurk et al., 2012^b,c,e,g,h,i^ (10), Ye et al., 2016^b,c,e^ (29)
	*w*MelPop	*Drosophila melanogaster*	*Aedes aegypti, Drosophila melanogaster*	FHV, IIV-6, YFV	Teixeira et al., 2008^a,b^ (6), Van den Hurk et al., 2012^b,c,e,h,i^ (10)
	*w*MelCS	*Drosophila melanogaster*	*Drosophila melanogaster*	Kallithea virus, La Crosse virus	Palmer et al., 2018^a^ (36), Glaser & Meola, 2010^b^ (12)
	*w*AlbB	*Aedes albopictus*	*Aedes aegypti, Culex tarsalis*	CHIKV, DENV, WNV	Ant et al., 2018^b^ (19)
	*w*AlbA	*Aedes albopictus*	*Aedes aegypti*	SFV	Ant et al., 2018^b^ (19)
	*w*AlbA + *w*AlbB	*Aedes albopictus*	*Aedes albopictus*	CHIKV, DENV	Mousson et al., 2010^b^ (37), Mousson et al., 2012^a,b,e^ (21)
	Male-killing *wD. bifasciata*	*Drosophila bifasciata*	*Drosophila bifasciata*	DCV, FHV	Longdon et al., 2012^a^ (38)
	*w*Bai	*Drosophila baimaii*	*Drosophila simulans*	DCV, FHV	Martinez et al., 2014^a,b^ (22)
	*w*Bic	*Drosophila bicornuta*	*Drosophila simulans*	DCV, FHV	Martinez et al., 2014^a,b^ (22)
	*w*Bor	*Drosophila borealis*	*Drosophila simulans*	DCV, FHV	Martinez et al., 2014^a,b^ (22)
	*w*Ha	*Drosophila simulans*	*Drosophila simulans*	DCV, FHV	Martinez et al., 2014^a,b^ (22), Martinez et al., 2017^b^ (23), Osborne et al., 2009^b^ (24)
	*w*Ri	*Drosophila simulans*	*Drosophila simulans*	DCV, FHV	Martinez et al., 2017^b^ (23), Osborne et al., 2009^b^ (24)
	*w*No	*Drosophila simulans*	*Drosophila simulans*	DCV, FHV	Martinez et al., 2017^a,b^ (23), Osborne et al., 2009^a,b^ (24)
	*w*Inn	*Drosophila innubila*	*Drosophila simulans*	DCV, FHV	Martinez et al., 2014^a,b^ (22)
	*w*Ma	*Drosophila simulans*	*Drosophila simulans*	DCV, FHV	Martinez et al., 2014^a,b^ (22), Martinez et al., 2017^b^ (23)
	*w*Pro	*Drosophila prosaltans*	*Drosophila simulans, Drosophila prosaltans*	DCV, FHV	Martinez et al., 2014^a,b^ (22), Martinez et al., 2017^b^ (23)
	*w*San	*Drosophila santomea*	*Drosophila simulans*	DCV, FHV	Martinez et al., 2014^a,b^ (22)
	*w*Sh	*Drosophila sechellia*	*Drosophila simulans, Drosophila sechellia*	DCV, FHV	Martinez et al., 2014^a,b^ (22), Martinez et al., 2017^a,b^ (23)
	*w*Tri	*Drosophila triauraria*	*Drosophila simulans, Drosophila triauraria*	DCV, FHV	Martinez et al., 2014^a,b^ (22), Martinez et al.2017^a,b^ (23)
	*w*Tei	*Drosophila teissieri*	*Drosophila simulans, Drosophila teissieri*	FHV	Martinez et al., 2017^b^ (23)
	*w*Yak	*Drosophila yakuba*	*Drosophila simulans*	DCV, FHV	Martinez et al., 2014^a,b^ (22)
	*w*Ana	*Drosophila ananassae*	*Drosophila simulans, Drosophila ananassae*	FHV	Martinez et al., 2014^a,b^ (22), Martinez et al., 2017^a,b^ (23)
	*w*Stv	*Drosophila sturtevanti*	*Drosophila simulans, Drosophila sturtevanti*	FHV	Martinez et al., 2014^a,b^ (22), Martinez et al., 2017^a,b^ (23)
	*wA. subalbatus*	*Armigeres subalbatus*	*Armigeres subalbatus*	JEV	Tsai et al., 2006^c^ (39)
	*w*Tro	*Drosophila tropicalis*	*Drosophila simulans, Drosophila tropicalis*	DCV, FHV	Martinez et al., 2014^b^ (22), Martinez et al., 2017^b^ (23)
	*w*Suz	*Drosophila suzukii*	*Drosophila suzukii*	DCV, FHV	Cattel et al., 2016^a,b,d^ (32), Martinez et al., 2017^a,b^ (23)
Increase in susceptibility	*w*Mel	*Drosophila melanogaster*	*Aedes aegypti*	Flavivirus OTU1, Flavivirus OTU2, Flavivirus OTU3, Flavivirus OTU20, Flavivirus OTU21	Amuzu et al., 2018^b^ (25)
	*w*Exe1	*Spodoptera exempta*	*Spodoptera exempta*	SpexNPV	Graham et al., 2012^a^ (40)
	*w*Ha	*Drosophila simulans*	*Drosophila simulans*	DCV	Martinez et al., 2014^b^ (22)
	*w*San	*Drosophila santomea*	*Drosophila simulans*	FHV	Martinez et al., 2014^b^ (22)

Study measured: a) host survival, b) viral titer, c) infection rate.

Result varied among: d) host genotype, e) infection/transmission/dissemination, f) days post infection, g) infection type (oral or intratoraxic), h) virus strain, i) viral titer inoculated in the host.

CHIKV, chikungunya virus; CrPV, cricket paralysis virus; CpVD, Culex pipiens densovirus; DCV, Drosophila C virus; DENV, dengue virus; FHV, Flock House virus; IIV-6, insect iridescent virus 6; JEV, japanese encephalitis virus, RRSV, rice ragged stunt virus SFV, Semliki Forest virus, SpexNPV, Spodoptera exempta nucleopolyhedrovirus; WNV, West Nile virus; YFV, yellow fever virus; ZIKV, zika virus.

For each Wolbachia strain tested we report if there was protection, no protection or increase in susceptibility to viral infection. We present the natural host species of the strains, the hosts species in which the strains were tested, and the virus that were tested in the hosts.

**Figure 1:**
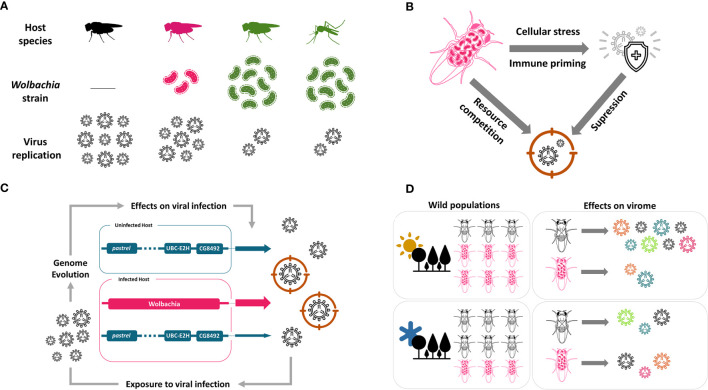
*Wolbachia* antiviral effect in insects. **(A)** *Wolbachia* protects insects against RNA viruses. The protection is dependent on *Wolbachia* density, which varies between strains. Strains can be experimentally transferred to new hosts, such as mosquitoes. **(B)** *Wolbachia* can activate host immune system in some cases, but the mechanism of defense can also be related to competition with virus for cellular resources. The specific mechanism is not yet known. **(C)** Host immune response fight against virus, but its action and evolution are slowed down in the presence of *Wolbachia*. Colored arrows and their width represent genome and its participation in antiviral effect, respectively. **(D)** Environmental conditions, as temperature, determine *Wolbachia* antiviral response. In hot climate, *Wolbachia* may have a more important role protecting the host, and this can lead to higher *Wolbachia* prevalence on hot climate regions. But it is not yet known if *Wolbachia* reduces the virome in wild insect populations. This figure is made in conjunction with icons provided by thenounproject.com. The icons are: “Bacteria” by farra nugraha; “Virus” by KonKapp; “Immune System” by Bartama Graphic; “Immunity” by Timofey Rostilov; “Forest” by ProSymbols; “Sun” by Alice Design; and, “Cold” by Landan Lloyd.

The level of protection against viruses varies among *Wolbachia* strains and depends on their density within the host (22, 42). It is common to transfer high density strains into new hosts, such as mosquitoes, to test for protection against viruses (**Figure 1A**). Thus, protection generally occurs in host-*Wolbachia* interactions that are not natural, but artificial (43). For example, the virulent strain *w*MelPop, originally isolated from *D. melanogaster* (44, 45), protects against different viruses in *Aedes aegypti* (**Table 1**). However, *w*MelPop is a strain that was identified only in laboratory and there is no record of it in nature. Other *Wolbachia* strains commonly used in experiments that have broad protection against viruses are *w*Mel, *w*MelCS, both isolated from *D. melanogaster*, *w*Au, isolated from *D. simulans*, *w*AlbB, isolated from *Aedes albopictus*, and *w*Stri, isolated from the planthooper *Laodelphax striatellus* (**Table 1**). Martinez and colleagues investigated antiviral protection in many *Wolbachia* strains originated from different *Drosophila* species after transfer into the same genetic background of *D. simulans*. Interestingly, they found that protection is not determined by host genotype, but by *Wolbachia* strain (23). All these studies showing that different strains protect different hosts against many RNA viruses were conducted in the laboratory, and there is still little evidence of the *Wolbachia* antiviral effect in nature (see last section below).

Another issue is that most studies that test for virus protection by *Wolbachia* are carried out using only the adult stage. So far, only Graham et al. (40) tested for viral protection in larval stages of *Spodoptera exempta*, and we still have no information of protection on pupae. Moreover, these results may be affected by the inoculation method in the laboratory. All studies in flies use systemic infection (stabbing or microinjection), while in mosquitoes some studies use oral infection besides microinjection. Although methods such as microinjection allow greater viral dose precision, we know that in nature insects acquire many pathogens by feeding (46, 47). Therefore, although there is a general pattern of protection against viruses in laboratory studies, there are some limitations on the methods used. Further studies testing *Wolbachia’s* antiviral protection in insect host using methods that approximate of how infections occur in nature, such as oral infection (46, 47), are essential to understanding the dynamics between *Wolbachia* and viruses in wild populations.

*Wolbachia* infects about 50% of all insect species (48), and we can hypothesize that the antiviral protection may be one of the reasons for *Wolbachia* being so widely spread among arthropods. However, studies on *Wolbachia*’s viral protection are still limited to flies and mosquitoes, with the exception of one study on a Lepidoptera host (40) and one study on a Hemiptera host (33). Thus, more studies on different insect families are essential to test if the antiviral effect also occurs in other insects, and how likely it may be one of the main reasons for the high prevalence of *Wolbachia* in natural insect populations.

## The Possible Mechanisms

Since the discovery of *Wolbachia* antiviral protection different mechanisms of action have been proposed, but up to now, there is no consensus on the underlying mechanism [reviewed by Lindsey et al (49)]. Current studies work on two main hypotheses to explain *Wolbachia* interference in viral replication: the activation of host immunity and competition with virus for cellular resources (**Figure 1B**).

The first hypothesis is that *Wolbachia* can directly activate innate immunity of the host prior to virus infection (immune priming), interfering with virus replication. The presence of the bacterium in host cells leads to cellular stress, including oxidative stress that activates host immune pathways (50). *Wolbachia* preactivates mosquito innate immunity by the oxidative stress, upregulating Toll pathway genes, known to be responsible for protection against dengue virus (8, 9, 50). Immune effector genes upregulation in *A. aegypti* suggests that the protection due to immunity priming is responsible for the viral interference (8, 9). However, the upregulation in the immune pathway genes is variable in different species and it seems to be influenced by the time of host-*Wolbachia* coevolution. For instance, there is no upregulation on Toll or IMD genes by *Wolbachia* in its native host *Aedes fluviatilis*, but other immune-related genes are indeed modulated, as oxidative stress-related genes (51). The generation of oxygen reactive species itself is an example of immune response that vary between novel and native host, ranging from triggering oxidative stress to redox homeostasis restoration [reviewed by Zug and Hammerstein (52)]. But there is evidence that *Wolbachia*-induced oxidative stress is involved in virus blocking both in transinfected mosquito and *Drosophila* with a natural *Wolbachia* infection (50, 53).

The second hypothesis is that resources shared by *Wolbachia* and the virus can represent a limitation for development of the latter when they are co-infecting their host. As discussed in the previous section, *Wolbachia* protects mainly against RNA viruses which depends on specific cellular resources, the integrity of intracellular membranes for replication, and the host translation apparatus for virus protein production (49). Any disturbance caused by *Wolbachia* on these cellular components presumably interferes with virus replication. For instance, depletion, reduction, or modification of certain host lipids affect virus replication (54, 55). In particular for cholesterol, providing or restoring its intracellular traffic recover virus replication in a *Wolbachia*-infected host, indicating both the role of cholesterol in virus development and *Wolbachia* interference in host lipid availability (55, 56). In another recent example, it was found that *Wolbachia* and virus have antagonistic effect in the host expression of *prat2*, a gene involved in nucleotide synthesis (57).

Additionally, several approaches have shown that antiviral protection occurs in host bearing high density of *Wolbachia*, with no detectable protection is host with low symbiont density (22, 24). The same result is obtained in experimental manipulation of *Wolbachia* density with antibiotics (58). The control of symbiont density is dependent on the symbiont genotype and, in the case of *Wolbachia* strains isolated from *D. melanogaster*, the genetic basis of density determination has been assigned to the Octomom region which presents several duplications, or a deletion of the entire region, in high-density symbionts (59–61). However, one recent study with controlled genetic background showed an intriguing example of *Wolbachia* with no antiviral action in *A. aegypti*, even in relatively high density (62). Other than density, host development stage and temperature seem to modulate *Wolbachia* antiviral properties (61, 63).

The mechanism behind *Wolbachia* antiviral protection became an active area of research. New experimental approaches, such as the forward genetic screens applicable on genetically intractable bacteria (61), are extremely promising to pursue this question. One example of how recent experimental advances can bring progress to long standing questions is the case of cytoplasmic incompatibility caused by *Wolbachia*. Cytoplasmic incompatibility has been studied since 1971, yet only recently its mechanism was uncovered (1, 64, 65). The cytoplasmic incompatibility is controlled by two phage WO genes, *cifAwMel* and *cifBwMel*, present in the *Wolbachia* genome (66). Similar advances are likely to figure out the specific antiviral mechanism in the following years.

## Influence on Evolution of Host “INTRINSIC” Immunological Resistance Mechanisms

Although *Wolbachia* confers viral protection to insects, natural insect populations have other means to fight against viruses (67, 68). Insects usually rely on the mechanisms of RNA interference, apoptosis, NF-κB pathways and translation control from its innate immune system to get along viral pathogens (69). Nevertheless, the population’s ability to resist the plethora of viruses present in nature lies on its standing genetic variation on these mechanisms or the sudden appearance of beneficial mutations (70). However, in the presence of *Wolbachia*, the extended mutualistic genotype could mask or even substitute host’s intrinsic mechanisms of antiviral defenses, shifting its adaptive landscape (71) (**Figure 1C**). Some recent experimental evolution studies have addressed how the presence of *Wolbachia* can alter the evolution of intrinsic antiviral mechanisms in insects.

In a pioneer study, Martins and colleagues used an experimental evolution approach in which *Drosophila melanogaster* populations were subjected to continuous DCV injections for a few generations (72). Compared with control populations that were not exposed to the virus, infected populations showed increased survival after DCV infection, and also increased survival after infection by cricket paralysis virus (CrPV) and FHV (72). This increased resistance to viral infection was associated with three candidate genes on the fly’s genome - *pastrel*, *Ubc-E2H* and *CG8492* (72). In another experimental evolution study, Martinez and colleagues directly tested how the presence of *Wolbachia* can alter evolution of intrinsic antiviral mechanisms (71). They focused on a polymorphism of the gene *pastrel* that explains most of the variation on DCV resistance in *D. melanogaster* populations (73, 74). They infected populations with and without *Wolbachia* for nine generations. Resistance to DCV and the frequency of the resistant *pastrel* allele increased in all populations exposed to the virus compared with virus-free control populations (71). Most interestingly, the frequency of the resistant *pastrel* allele after nine generations was lower in *Wolbachia* infected populations than in the symbiont-free populations. After experimentally removing *Wolbachia*, the populations that had *Wolbachia* during the selection experiment was much less resistant to the virus than the *Wolbachia*-free populations. This experiment shows that the presence of *Wolbachia* resulted in weaker selection on the host intrinsic antiviral defenses, making the host addicted to the protection caused by the symbiont (71). Another study showed that DCV infection selected for a particular *Wolbachia* strain that enhances survival and fecundity in the presence of DCV (75). Finally, Faria and colleagues showed that intrinsic antiviral defenses can replace symbiont protection (72, 76). They used previously selected populations for increased virus resistance (72), and removed the symbiont from these populations. They first observed a severe drop in survival after DCV infection, but resistance significantly increased in subsequent generations reaching the same levels as seen in the presence of *Wolbachia* after 20 generations (76).

These studies show that *Wolbachia* can change the strength of selection on host antiviral mechanisms, leading to evolutionary addiction (71, 72, 75, 76). Because *Wolbachia* prevalence varies in natural populations, this may be one mechanism that maintains genetic variation in intrinsic antiviral resistance in populations (76). One interesting interplay is that different *Drosophila* clades respond differently to viral infections (77), therefore, variation in resistance and susceptibility of hosts could be mirrored by the success and establishment of *Wolbachia* in some clades but not others in nature (78). In addition, it would be remarkably interesting to investigate how the presence of *Wolbachia* in some clades may affect the evolution of host-shifts by viruses (79).

## Importance in Wild Populations

The *Wolbachia* antiviral effects were intensely studied in the last decade because of its importance in the field of public health. However, their ecological importance in wild populations has rarely being addressed. Around 50% of insect species may carry one or more strains of *Wolbachia* (48), meaning that almost 3 million insect species are infected. Therefore, *Wolbachia* may be a major component of antiviral defenses in nature (43). But just recently some studies started to test if *Wolbachia* can confer protection against viruses in wild insect populations. The antiviral effects of *Wolbachia* may mean that in nature it is frequently a mutualist that protects its host against infection. This may explain why *Wolbachia* strains that do not cause cytoplasmic incompatibility and have no obvious phenotypic effect can invade and be maintained in populations (80). Theory predicts that cytoplasmic incompatibility can only invade when local infection frequencies becomes sufficiently high to offset imperfect maternal transmission and infection costs (81, 82). However, recent data suggested that *Wolbachia* can spread from arbitrarily low frequencies (80). In this scenario, there appears to be a fitness advantage for the host caused by *Wolbachia* in natural populations (83). This fitness advantage may be *Wolbachia* antiviral effects. This is expected by the studies carried out in the laboratory showing the antiviral effect, but just now some studies started to test this in wild populations. It is interesting to notice that *Wolbachia* can also protect insects against bacteria and entomopathogenic fungi (84–86), and this can also add to the possible mutualistic effect in natural populations.

*Drosophila* flies have been used as the main model to study insect virus interactions, but until recently we knew extraordinarily little about the virus community that infect wild *Drosophila* populations. This is changing rapidly with recent studies using metagenomic approaches (87). In 2015, Webster and colleagues used metagenomic techniques in more than 2000 wild collect *Drosophila melanogaster* flies and discovered more than 20 new viruses (41). They found a high prevalence of virus infection with more than 30% of the wild collected individuals carrying a virus. There was also large variation in prevalence among the 17 sampled locations across the world. Because *Wolbachia* prevalence in these locations varied from 1.6% to 98% - with a mean of about 50% - they tested for associations between the prevalence of *Wolbachia* and the different viruses among and within populations. They could not find any association, indicating that *Wolbachia* is not an important determinant of virus incidence in the wild (41). However, as pointed by the authors, they had a small sample size per population resulting in low statistical power to detect an association. In addition, they looked only on the effect of *Wolbachia* on prevalence, but *Wolbachia* can also be influencing virus titer on infected flies.

In 2018, Shi and colleagues tested the effect of *Wolbachia* on viral abundance on six *D. melanogaster* populations sampled in Australia (88). They first sequenced total transcriptome of pools of *Wolbachia*-infected and *Wolbachia*-free lines to estimate viral abundance. Despite finding high RNA virus’ abundance in all pools, they did not find any *Wolbachia* protective effect. They also sequenced the transcriptome of individual *Wolbachia*-infected and *Wolbachia*-free flies from one location, but again did not find any *Wolbachia* protective effect (88). These results should be interpreted with caution as well, since they sequenced only 122 flies in the pools, plus 40 individual flies. Given the large variation among pools in viral abundance and in the prevalence that varied from two to five viruses per pool, the statistical power to detect an effect was low. Additionally, they did not sequence wild collected flies, but F1 or F3 of laboratory cultured lines that were kept at 19°C. Unfortunately, it was discovered, very recently, that the antiviral effect of the *Wolbachia* strain wMel in *D. melanogaster* depends on temperature (63). The strong protection observed when flies develop from egg to adult at 25°C is greatly reduced or disappear when flies develop at 18°C (63). Therefore, the development conditions used by Shi et al. may have masked any possible *Wolbachia* protective effect.

Interestingly, the recent study on the effect of temperature on the *Wolbachia* antiviral effect (63) offers a hint on this puzzle. It is interesting that the *Wolbachia* antiviral effect observed at high development temperature is extremely reduced when flies develop at low temperatures. This was observed with different genotypes of *D. melanogaster*, different *Wolbachia* lineages, and different viruses, suggesting this is a general phenomenon (63). These results suggest that in nature the mutualistic effect of virus protection will vary geographically and seasonally depending on climate, and this will result in the prevalence of *Wolbachia* being higher in tropical regions (**Figure 1D**). This is indeed what is observed in nature, where the frequency of *Wolbachia* is generally higher in populations from tropical regions (89). This pattern, although only a correlative suggestion, indicates that the antiviral protection may be the mutualistic effect in natural populations responsible for the widespread success of *Wolbachia*.

## Conclusions

Since the *Wolbachia* antiviral effect in insects was discovered 12 years ago (6, 7), researchers have intensely studied this phenomenon. *Wolbachia* has even been successfully used to control the prevalence of human arboviruses, such as dengue, in mosquito populations (16, 17, 90). We learned a lot about the basic biology of the host-*Wolbachia*-virus interaction, but there are still many knowledge gaps. We now know the antiviral effect depends on *Wolbachia* strain, with only high-density strains having the antiviral effect. However, it is still unknown whether the antiviral effect occurs in insect species other than mosquitoes, flies and a planthopper. Importantly, the specific mechanism underlying antiviral protection has not been fully elucidated; upregulation of the host immune system or competition between *Wolbachia* and RNA viruses inside the host cell for some yet unknown resource necessary for virus replication are likely hypothesis (49, 52, 56). We have also learned that *Wolbachia* can alter the intensity of selection on host antiviral defenses, making the host more dependent on the symbiont for protection (71). We still do not know if the antiviral effect occurs in natural populations of insects and if it is the major mutualistic effect responsible for the extremely high prevalence of *Wolbachia* in insects. If it does, *Wolbachia* may be a major component of antiviral defense in nature.

## Author Contributions

AP, CC, MM, and RC wrote the paper. All authors contributed to the article and approved the submitted version.

## Funding

Funding for this work was provided by São Paulo Research Foundation (FAPESP) (2013/25991-0, 2015/08307-3, 2018/01295-8 and 2019/03997-2), CNPq (154568/2018-0, 307015/2015-7 and 307447/2018-9), and a Newton Advanced Fellowship from the Royal Society.

## Conflict of Interest

The authors declare that the research was conducted in the absence of any commercial or financial relationships that could be construed as a potential conflict of interest.
